# Stimulant-Induced Pituitary Failure and Reversible Azoospermia

**DOI:** 10.7759/cureus.14269

**Published:** 2021-04-03

**Authors:** Tori E Abdalla, Daniela Kotsonis, Jordan Best, Ranjith Ramasamy, Ellen Wood

**Affiliations:** 1 Biomedical Sciences, Philadelphia College of Osteopathic Medicine, Philadelphia, USA; 2 Research, Boston University, Boston, USA; 3 Medical Education, Nova Southeastern University, Miami, USA; 4 Urology, University of Miami, Miami, USA; 5 Reproductive Endocrinology and Infertility, IVFMD, Cooper City, USA

**Keywords:** infertility, amphetamine-dextroamphetamine, azoospermia, stimulant, methylphenidate

## Abstract

Attention-deficit hyperactivity disorder (ADHD) is a commonly diagnosed disorder that is managed with stimulant medications, which function by increasing the levels of dopamine in the brain. Excess dopamine has been known to affect several body systems, including the endocrine system. This case presents male factor infertility caused by a negative interaction between excess dopamine and the endocrine system, inducing pituitary failure, which led to azoospermia. The patient and her partner presented to the fertility clinic for evaluation after one year of failing to conceive. The patient’s partner had been treated throughout the conception of their first three pregnancies for ADHD with methylphenidate (Ritalin) for many years; however, eight months prior to presentation at the clinic, the partner had been switched to amphetamine-dextroamphetamine (Adderall) for treatment of ADHD. A fertility evaluation revealed azoospermia which was confirmed via two separate semen analyses two weeks apart. In addition, the patient’s total testosterone, prolactin, luteinizing hormone, and follicle-stimulating hormone were below normal limits. A normal semen analysis was obtained after a five-month withdrawal of amphetamine-dextroamphetamine, which was followed by a naturally conceived pregnancy. The possibility of pre-testicular azoospermia caused by medication-induced pituitary failure should be considered in males prescribed stimulant medication who are seeking to reproduce.

## Introduction

Attention-deficit hyperactivity disorder (ADHD) is the most commonly diagnosed neuropsychiatric disorder and can persist throughout adulthood [1]. Stimulant medications have been found to manage ADHD with two popular treatments being amphetamine-dextroamphetamine (Adderall) and methylphenidate (Ritalin) [2]. Both medications work similarly by increasing catecholamine transmission and inhibiting the reuptake of dopamine and norepinephrine. However, there are two marked differences between the medications. They act on different neuronal pools of catecholamines: amphetamine-dextroamphetamine acts on newly synthesized dopamine, while methylphenidate acts on storage granules [2]. The second difference is between the increase in cytosolic dopamine because while methylphenidate keeps dopamine sequestered, amphetamine-dextroamphetamine releases dopamine from synaptosomes [2].

With the amphetamine-dextroamphetamine-induced dopamine increase released from synaptosomes, unwanted effects can arise, such as the inhibition of gonadotropin-releasing hormone (GnRH) neuronal excitability [3]. GnRH neurons are responsible for the gonadotropin cascade, and subsequently, the release of luteinizing hormone (LH) and follicle-stimulating hormone (FSH), two critical hormones within the reproductive system. With the increase in dopamine-inhibiting GnRH neurons, there is a subsequent decrease in levels of LH and FSH. Reduced LH level has been shown to affect spermatogenesis, specifically in a study concluding that infertility arises in LH knockout mice [4]. LH stimulates Leydig cells whose primary role is to produce testosterone [4]. Testosterone is a crucial hormone for the initiation and maintenance of spermatogenesis. Therefore, in the absence of LH, Leydig cells are not stimulated, creating a decrease in testosterone and negatively affecting spermatogenesis. The physiology of the gonadotropin cascade in conjunction with the mechanism of action of stimulant medication may lead to decreased LH production and subsequent azoospermia.

In this article, we review the case of a man who naturally conceived three pregnancies with his partner while taking methylphenidate to becoming azoospermic after switching to amphetamine-dextroamphetamine.

## Case presentation

A 39-year-old G3 P2 (G = gravida = total pregnancies, P, parity = live births) with a obstetric history of two prior uncomplicated cesarean sections and one spontaneous abortion presented to the fertility clinic for evaluation after not being able to conceive for one year since her pregnancy loss. Her medical history was unremarkable other than cryosurgery for a lesion on the cervix nine years ago. Her medications included prenatal vitamins and clomiphene citrate for the past two months prescribed by her gynecologist. All her prior pregnancies were conceived naturally without medical intervention. Her partner’s history was significant for ADHD and bipolar disorder. At the time of presentation, he was being treated with amphetamine-dextroamphetamine 120 mg, risperidone, and buprenorphine. He was started on amphetamine-dextroamphetamine eight months ago and his previous medication, methylphenidate, was discontinued.

A complete evaluation for female and male factor infertility including hormonal blood work such as FSH to evaluate egg quality, a recurrent pregnancy loss panel, which included screening for genetic factors and antiphospholipid syndrome, a hysterosalpingogram, and a semen analysis was ordered. All blood testing for the female partner returned completely normal and the hysterosalpingogram showed patent Fallopian tubes. The semen analysis came back azoospermic, confirmed by centrifuge. As this finding was not consistent with the patient’s history, a repeat semen analysis was ordered two weeks later, confirming the finding of azoospermia.

The patient’s spouse was then referred for a hormonal as well as a urological evaluation. The hormonal evaluation came back with FSH of less than 0.7 mIU/mL (1.6-8.0 mIU/mL), LH less than 0.2 mIU/mL (1.5-9.3 mIU/mL), a total testosterone of 84.0 ng/dL (250-827 ng/dL), and a prolactin of 2.0 ng/mL (2.0-18.0 ng/mL). Estradiol was normal at 43.0 pg/mL (more than 29 pg/mL). The patient denied any history of head trauma or significant illness with a high fever in the past year. The patient was subsequently sent for an magnetic resonance imaging (MRI) of his pituitary gland which came back normal. He was then referred to a medical endocrinologist for evaluation of his pituitary failure.

The medical endocrinologist recommended using fertility vitamins for the patient’s spouse, as well as discontinuing the use of amphetamine-dextroamphetamine. The patient then returned to the fertility clinic for a repeat semen analysis five months after discontinuing his amphetamine-dextroamphetamine. The semen analysis now revealed a total sperm count of 120 million, gross motility of 65%, and morphology normal forms of 26%.

One month later, the patient successfully conceived; however, this pregnancy ended in another spontaneous loss due to a chromosomal abnormality. Three months later, the patient conceived again via ovulation induction treatment with intrauterine insemination (IUI). Both IUI samples were over 10 million total motile count. The patient subsequently conceived again and delivered a healthy full-term baby girl.

## Discussion

This case depicts a couple that naturally conceived three pregnancies and then appeared to the clinic due to infertility while trying to conceive for the fourth time. After evaluation of both partners, findings revealed that the male partner was azoospermic due to clear pituitary failure on his hormonal evaluation. An MRI revealed that the hypothalamus and the pituitary gland were normal. Due to a recent medication change, it was recommended that the male partner discontinue his new stimulant medication, amphetamine-dextroamphetamine. After five months of discontinuing amphetamine-dextroamphetamine, his semen analysis was normal and the couple was able to naturally conceive. This finding indicated that the ADHD medication amphetamine-dextroamphetamine contributed to the male patient’s pituitary failure and infertility. The male patient’s other medications, risperidone and buprenorphine, were not considered to cause his infertility due to improved semen analysis parameters five months after the discontinuation of amphetamine-dextroamphetamine.

Male factor infertility can result from several different conditions, obstructive or non-obstructive, that can interfere with spermatogenesis, decreasing sperm production and negatively affecting sperm parameters, leading to azoospermia [5]. Azoospermia diagnosis is categorized into three categories: pre-testicular, testicular, or post-testicular. A testicular azoospermic disorder is considered a primary failure, while pre-testicular refers to secondary testicular failure in regard to endocrine disorders, such as pituitary failure [5]. Pre- and post-testicular failure are both recognized as generally correctable disorders [5]. In this specific case, the patient’s two semen analyses revealed azoospermia. A hormonal evaluation was conducted and found significantly decreased levels of FSH, LH, testosterone, and prolactin. The conclusion was made that pre-testicular failure contributed to the patient’s azoospermia. Due to the clear MRI and decreased hormone levels, the patient’s medical history was investigated which was significant for a medication switch that occurred between the couples’ first three naturally conceived pregnancies and infertility encountered while trying to conceive for a fourth time. The recommendation that the patient discontinue the amphetamine-dextroamphetamine successfully reversed the azoospermia, revealing the cause of the pre-testicular failure as stimulant medication induced.

Amphetamine-dextroamphetamine and methylphenidate are stimulant medications that increase the levels of dopamine within the body. However, amphetamine-dextroamphetamine releases the produced dopamine into the extracellular cytosol of the brain, while methylphenidate keeps the dopamine sequestered within the synaptosomes [2]. We hypothesized that the difference in mechanisms used by amphetamine-dextroamphetamine compared to methylphenidate was the reason for the male partner’s pituitary failure and the subsequent azoospermia. In research conducted by LeBlanc et al., the effects of dopamine infusions were studied in relation to hyperprolactinemia [6]. The study revealed that the dopamine infusion caused a significant drop in LH [6]. After the immediate effect of the dopamine infusion dissipated, the LH levels rebounded well [6]. This rebound could explain the reversible effects of amphetamine-dextroamphetamine seen on the patient’s third semen analysis, which revealed normal sperm parameters after medication discontinuation. LH is a gonadotropin that has the primary function to stimulate testosterone production, which is a primary hormone in the stimulation of spermatogenesis [7]. Dopamine decreases the excitability of GnRH-releasing neurons, inhibiting the gonadotropin cascade and causing a large decrease in LH levels [3,6]. A drop in LH levels can subsequently cause a decrease in testosterone, an important hormone driving spermatogenesis, and therefore, affecting the sperm produced.

The pharmacologic differences between amphetamine-dextroamphetamine and methylphenidate are believed to have affected the patient’s ability to naturally conceive with his wife. Known side effects of amphetamine-dextroamphetamine do not list male infertility or azoospermia. In 2005, the National Toxicology Program conducted an evaluation for the potential adverse effects of amphetamines on development and reproduction. No data on human reproductive toxicity were available and animal studies regarding methamphetamine were deemed insufficient [8]. No current literature examines the effects of prescribed stimulants on male spermatogenesis; however, prior studies have been conducted regarding amphetamine use and testosterone production, in addition to methamphetamine use and sperm production [9]. Both in vitro and in vivo studies have been completed revealing that amphetamine decreases testosterone production, leading researchers of a 1996 study, included in a 2019 systematic review regarding the relationship between substance abuse and male hypogonadism, to hypothesize that amphetamines could act on Leydig cells in a dose-dependent manner [9].

Although methamphetamine is an illegal and dangerous chemical compound that differs from prescribed high doses of amphetamine-dextroamphetamine, it potentially has similar effects on spermatozoa morphology and motility, in addition to steroid hormones within the body [10]. With an increase in dosage of methamphetamine in rats, a decrease in normal sperm parameters, such as concentration, motility, and morphology, was noted in a dose-consistent manner [10]. A dose-dependent relationship was also found between methamphetamine and increased apoptosis in rat spermatogonia and primary spermatocytes, creating a decrease in overall proliferation/apoptosis ratio in rats [9]. Methamphetamine intake and levels of testosterone, LH, and FSH have been studied in men with chronic methamphetamine use. The study revealed that the amount of methamphetamine consumption does affect the level of LH and testosterone, while not showing a clear correlation between FSH and methamphetamine [11].

A systematic review of the effects of ADHD medication looked at 17 animal studies conducted by Danborg et al. [12]. Out of the 17 studies, one focused on amphetamine and did not note any difference on ejaculation in mice, while other ADHD medications such as clonidine and methylphenidate showed impairment in animal reproduction due to ejaculation [12]. The 2017 review notes that there is a lack of studies on ADHD medication and reproduction impairment in humans. Some studies have focused on the association between increased dopamine and hormone impairment [6,7]; however, more research is needed focusing on the effects of stimulant medication in human reproduction, specifically in men.

## Conclusions

This case report offers insight into stimulant medication-induced pituitary failure that led to azoospermia and the subsequent reversal following discontinuation of medication. Despite not being mentioned as a side effect of amphetamine-dextroamphetamine, this report offers a case depicting the negative, but reversible, effect on gonadotropins and spermatogenesis that stimulant medications, specifically amphetamine-dextroamphetamine, can pose. There are studies focused on the effects of similar medications in animals and a few studies regarding similar medications in human development; however, the detrimental effects on fertility that could be linked to stimulant medication in humans, specifically males, has not been fully investigated. We recommend that the effects of stimulant medication on fertility, and the variation in effects based on duration of use and the use in conjunction with other medication, be further explored.
